# Evaluation of 3-Dimensional accuracy of guided implant placement using guided osteoperiosteal flap versus guided ridge splitting for atrophic maxillary augmentation (a randomized clinical trial)

**DOI:** 10.1186/s12903-025-07364-6

**Published:** 2025-12-05

**Authors:** Omar Ahmed ElSayed Hassan Salem, Ahmed Salah Elmahlawy, Mohamed Elsayed Saber

**Affiliations:** Oral and Maxillofacial Surgery Department, Faculty of Dentistry, Alexandria University, Alexandria, Egypt

**Keywords:** 3D printing, Accuracy, Digital workflow, Guided implant surgery, Osteoperiosteal flap, Piezosurgery, Ridge splitting, Static guide

## Abstract

**Background:**

Implant placement in atrophic ridges presents a clinical challenge for surgeons and the results are often unpredictable. Static guided surgery is well-documented in cases where bone is adequate in height and width. However, its application with simultaneous augmentation procedures - such as ridge splitting or osteoperiosteal flap - remains underexplored. This study evaluated three-dimensional accuracy of static guided implant placement after guided ridge splitting or osteoperiosteal flap elevation, using a fully digital, dual-guide surgical protocol.

**Methods:**

A total of 18 patients requiring horizontal ridge augmentation received 40 dental implants using a dual-guide static protocol. The first surgical guide was used for piezoelectric ridge splitting or osteoperiosteal flap elevation; the second allowed fully guided implant placement. Surgical guides were designed via cone-beam computed tomography (CBCT) and intraoral scan superimposition using BlueSkyPlan^®^ and in-office 3 Dimensional printing. Accuracy was assessed by comparing planned and actual implant positions on postoperative cone-beam computed tomography scans, measuring linear and vertical deviations at the entry point and apex, and angular deviation. Descriptive and inferential statistics were used to evaluate accuracy and subgroup differences.

**Results:**

All 40 implants were successfully placed without complications. All implants achieved primary stability and had uneventful healing. Both approaches provided high placement accuracy comparable with values reported in the literature. Comparison between ridge splitting and osteoperiosteal flap techniques revealed no statistically significant differences across coronal, apical, and angular deviation parameters. The osteoperiosteal flap group showed numerically smaller deviations, but the difference was not statistically significant (*p* > 0.05).

**Conclusions:**

A fully digital dual-guide static protocol enables accurate implant placement in augmented ridges using guided ridge splitting or osteoperiosteal flap techniques. This digital workflow may serve as a reliable alternative to conventional grafting methods in anatomically compromised sites.

**Trial registration:**

This Randomized Clinical Trial has been retrospectively registered at Clinical Trials.gov with identification number: NCT07103577 on 2025-07-29.

## Introduction

Following tooth extraction changes in alveolar bone morphology occur, particularly in anterior maxilla, affecting both horizontal and vertical ridge dimensions [1].

To counteract this resorption, augmentation is sometimes needed. Autogenous bone grafts remain the gold standard in augmentation procedures due to their osteogenic properties and greater predictability compared to allografts or xenografts, especially in cases with large defects [2].

Ridge splitting is one of the most commonly used augmentation procedures. This technique is used to horizontally augment the edentulous ridge either to enable implant placement or to accommodate interpositional bone grafts. This approach is indicated when sufficient vertical bone height is present for implant placement with no vertical bone defects [3]. One advantage of ridge splitting technique, compared to conventional grafting, is the ability of simultaneous implant placement. However, a major drawback is the risk of intraoperative complication of buccal bone fracture that may exacerbate the bone defect and result in additional bone loss during the procedure [4, 5].

An alternative to ridge splitting is the osteoperiosteal flap technique which involves out-fracturing the buccal plate while preserving the overlying labial soft tissue by avoiding flap reflection, maintaining the blood supply to the buccal bone. This approach is superior for both its ease and its biological benefits, by avoiding reflection of the mucoperiosteum, the blood supply to the outer cortical plate remains intact, sustaining bone viability and reducing the risk of postoperative remodeling. Additionally, this technique eliminated the need of a second bone harvest site [6–11].

Static computer-aided implant surgery (sCAIS) has improved surgical precision by integrating cone-beam computed tomography (CBCT), intraoral scans and prosthetically driven planning software This workflow permits the fabrication of 3D-printed surgical guides, which facilitate the placement of implants [12, 13]. Systematic reviews report that sCAIS provides better placement accuracy compared with freehand techniques, limiting angular, coronal, and apical deviations during final implant insertion [14–16].

However, even with guided systems, deviations may still occur due to mechanical tolerance in the sleeves and drills, surgical guide seating discrepancies, and operator technique [17–19].

Performing guided ridge augmentation techniques is frequently used to enable implant placement in atrophic ridges. However, these techniques disrupt cortical continuity and can compromise the stability and fit of surgical guides, leading to reduced implant placement accuracy [20, 21].

Recently, new protocols have been introduced that merge digital planning with ridge augmentation and implant placement, enabling the use of 3 dimensional (3D) printed surgical guides for both the osteotomy vector and implant placement in a single stage surgery [22, 23].

While techniques like guided ridge splitting, osteoperiosteal flaps, digital planning, sCAIS, ridge augmentation, piezosurgery, and PRF use have each been studied on their own.

This clinical trial integrated all of them into a single digitally guided protocol testing the impact of using two separate static guides—one for ridge splitting or osteoperiosteal flap elevation and another for implant placement—on (3D) implant accuracy.

## Materials and methods

### Study design and ethical approval

**Fig. 1:**
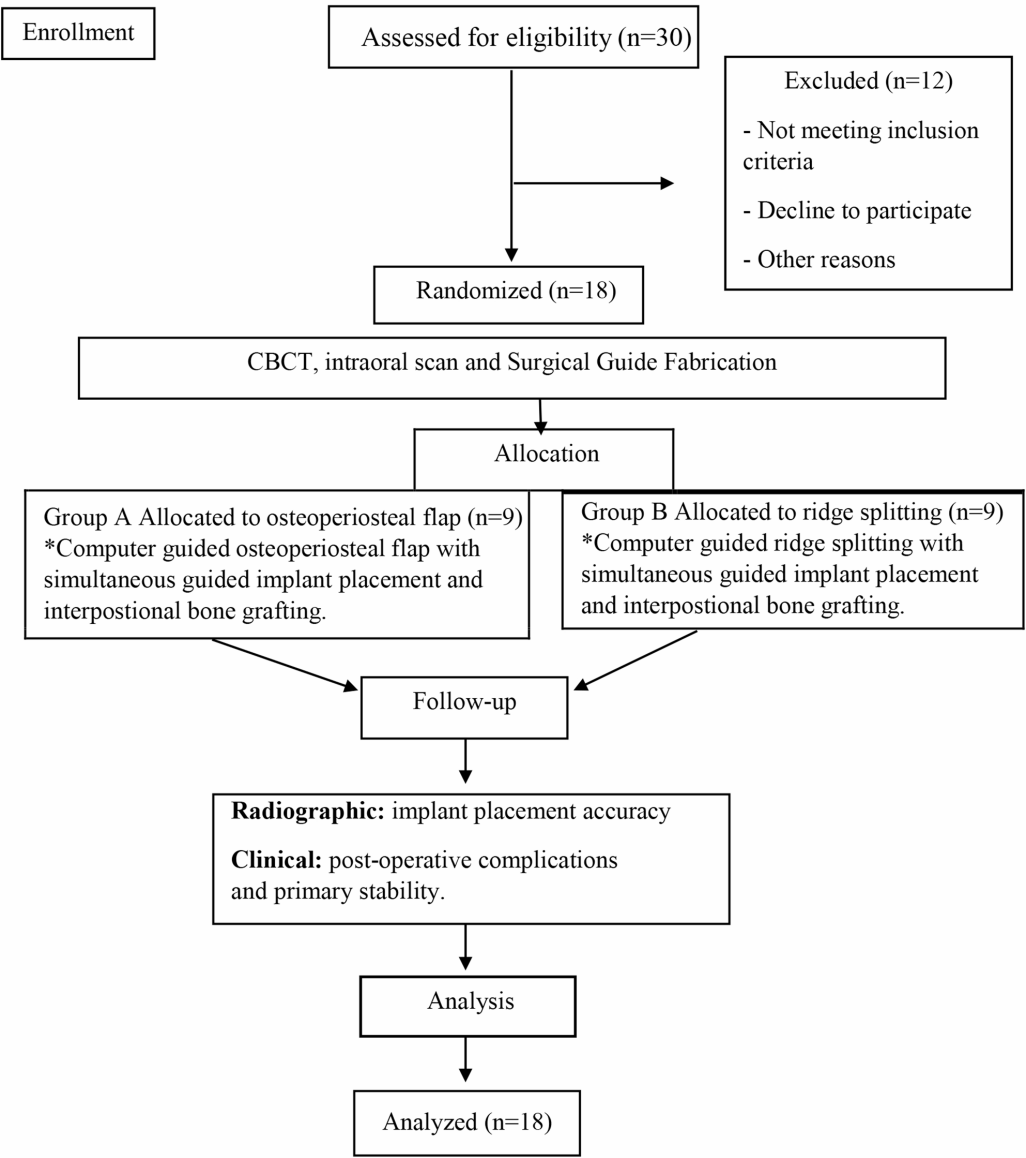
CONSORT flow diagram illustrating patient enrollment, randomization, intervention allocation, follow-up, and analysis for both the osteoperiosteal flap and ridge splitting groups

This randomized clinical trial has been retrospectively registered at Clinical Trials.gov with identification number: NCT07103577, 2025-07-29. It was approved by the Research Ethics Committee, Faculty of Dentistry, Alexandria University, Egypt (IRB No.00010556—IORG 0008839; ethics committee number: 0840–01/2024; date of approval: 2024–01–15). This study was conducted at the Department of Oral and Maxillofacial Surgery, Faculty of Dentistry, Alexandria University. Written informed consent was obtained from all patients prior to participation. This study followed the CONSORT guidelines and the principles of the Declaration of Helsinki [24, 25]. (Fig. 1)

### Sample size calculation

Sample size was calculated based on previously published data evaluating the accuracy of static guided implant placement. Assuming a mean linear deviation of 1.2 mm at the implant entry point with a standard deviation of 0.5 mm, a sample of 36 implants was determined to be sufficient to detect a clinically significant difference of 0.4 mm with 80% power and a 5% significance level (α = 0.05). This threshold was selected as deviations greater than 0.3–0.5 mm have been reported to impact the accuracy of prosthetically driven implant placement [12, 14, 16]. The calculation was performed using Rosner’s method [26] and G*Power (version 3.1.9.7) [27]. A total of 40 implants were ultimately included in the final analysis, slightly increasing the statistical power of the study.

### Randomization and blinding

A total of 18 patients were randomly allocated into 2 equal groups, group A (ridge splitting) and group B (osteoperiosteal flap). Randomization was done using a computer-generated list of random numbers created with Microsoft excel software (version 2019; Microsoft Corporation). The allocation concealment was done using sequentially numbered sealed envelopes. Three days before the intervention, each envelope was opened to identify the patient’s group allocation, allowing time for surgical guide fabrication.

### Patient selection

Inclusion criteria were: adults aged 20 to 45 years, missing anterior maxillary teeth, residual bone height of 8 to15 mm and bone width of 3 to 4 mm, adequate keratinized tissue, and good oral hygiene [28]. Exclusion criteria included uncontrolled diabetes [29], coagulation or immunologic disorders [30, 31], history of radiotherapy or bisphosphonate use, parafunctional habits, and heavy smoking (>25 cigarettes/d) [32].

### Preoperative planning

Cone-beam computed tomography (CBCT) scans were obtained using CS 9000 3D system (Carestream Health Inc., Rochester, NY, USA) along with intraoral digital scans acquired using Omnicam intraoral scanner (Dentsply Sirona, Bensheim, Germany).

Digital Imaging and Communications in Medicine (DICOM) files from the CBCT scans and Standard Tessellation Language (STL) models from the intraoral scans were imported into BlueSkyPlan software (version 4; BlueSkyBio, LLC, Grayslake, IL, USA) for implant planning and surgical guide fabrication.

**Fig. 2:**
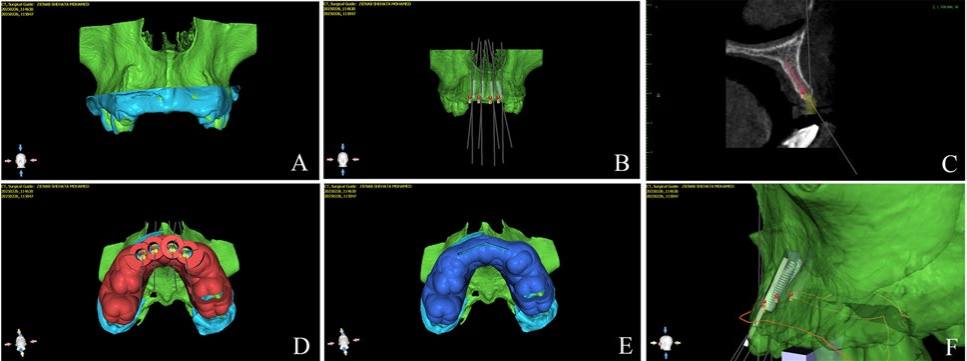
Preoperative digital treatment planning for a ridge splitting case. **A** CBCT segmentation and superimposition with intraoral scan. **B** Prosthetically driven implant planning. **C** Bone width measurement and implant size selection. **D** Implant placement surgical guide design. **E** Ridge splitting surgical guide design. **F** Osteotomy vector determination. CBCT = Cone Beam Computed Tomography

**Fig. 3:**
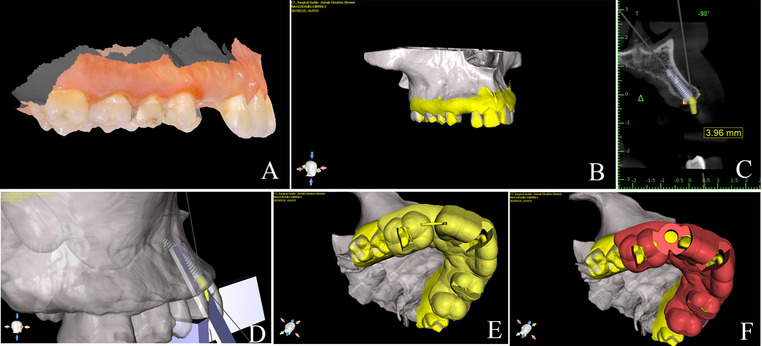
Preoperative digital treatment planning for an osteoperiosteal flap case. **A** STL file of intraoral scan. **B** CBCT segmentation and superimposition with intraoral scan. **C** Bone width measurement and implant size selection. **D** Osteotomy vector determination. **E** Osteoperiosteal flap surgical guide design. **F** Implant placement surgical guide design. CBCT = Cone Beam Computed Tomography; STL = Standard Tessellation Language

A dual-guide protocol was digitally designed:

- Surgical guide 1 ridge splitting or osteoperiosteal flap used to dictate piezoelectric osteotomies.
- Surgical guide 2 used for fully guided implant placement. (Figures 2 and 3)

Surgical guides were printed using a desktop 3D printer (Photon Mono X; Anycubic, Shenzhen, China) and post-processed with isopropanol rinsing, ultraviolet curing, and sterilized using 0.12% chlorhexidine immersion prior to surgery.

### Surgical procedure

All surgeries were performed under local anesthesia with 4% articaine and 1:100,000 epinephrine.

#### Group A (Ridge splitting Group)

**Fig. 4:**
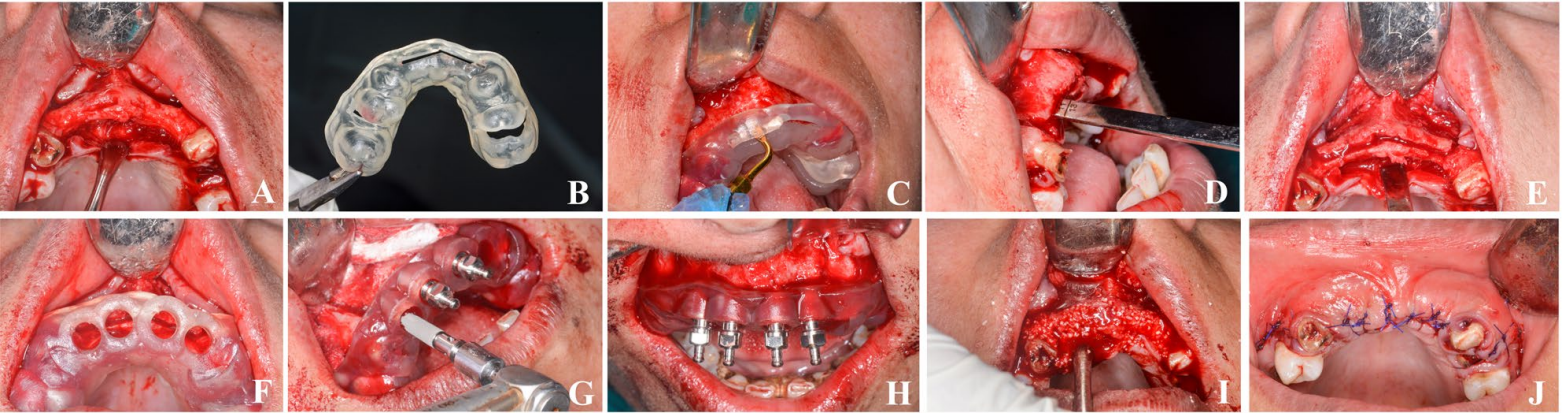
Guided ridge splitting with simultaneous guided implant placement. **A** Full-thickness mucoperiosteal flap reflection. **B** Tooth- and bone-supported ridge splitting guide. **C** Guided ridge splitting using piezosurgery. **D** Confirming osteotomy depth using graded osteotome. **E** Vertical osteotomy cuts. **F** Tooth- and bone-supported implant placement guide. **G–H** Fully guided implant placement. **I** Interpositional gap grafted with PRF mixed with xenograft. **J** Flap release and wound closure. PRF = Platelet-Rich Fibrin

A crestal incision with 2 vertical releasing incisions was followed by full mucoperiosteal flap elevation. The same guided piezoelectric osteotomy protocol was followed. (Fig. 4)

#### Group B (Osteoperiosteal flap Group)

**Fig. 5:**
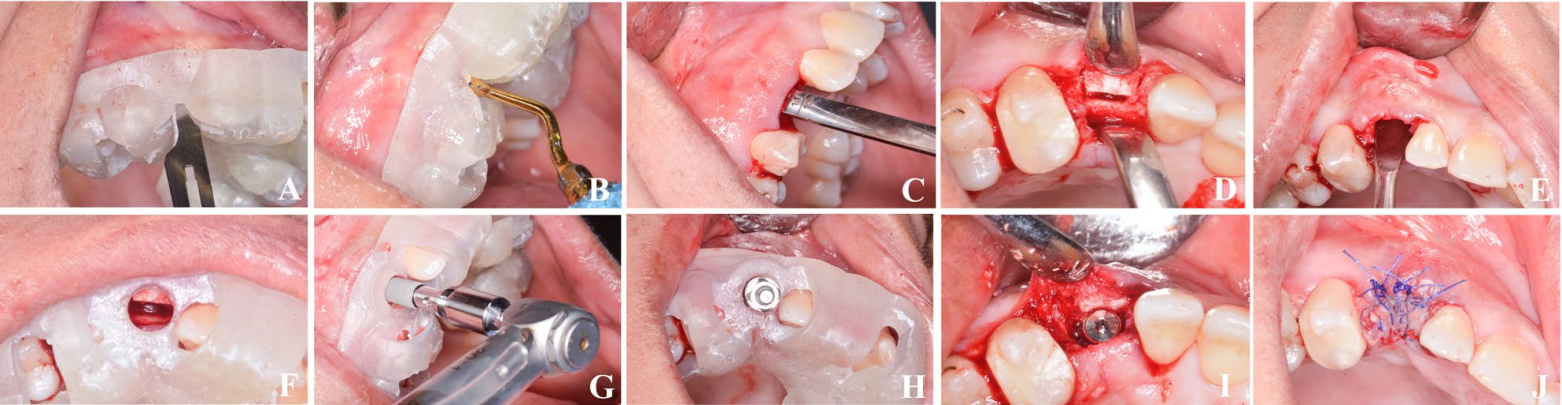
Guided osteoperiosteal flap with simultaneous guided implant placement.** A** Guided crestal incision using surgical guide. **B** Osteoperiosteal flap elevation using piezosurgery. **C–D** Confirming osteotomy depth and vertical cuts using osteotome. **E** Mobilization of the buccal plate. **F–H** Guided implant drilling and placement. **I** Ridge width increase with preserved buccal plate. **J** Wound closure

A paracrestal incision was made. The osteoperiosteal flap surgical guide was stabilized and used to direct piezoelectric osteotomies (Surgic Touch; Guilin Woodpecker Medical Instrument Co., Guilin, China; tip US2) to a depth 3 mm short of planned implant length. Two vertical osteotomies (1.5–2 mm from adjacent roots) were made to facilitate greenstick fracture of the buccal plate. Osteotomes (Dentium Co., Ltd., Seoul, South Korea) were used for controlled lateral displacement while preserving periosteal attachment. (Fig. 5)

In both groups, implant osteotomies were prepared using a fully guided protocol, and implants (Dentcon^®^ Dental Implant Systems, Ankara, Turkey) were placed 2 mm subcrestally using the second guide. Primary stability was assessed with resonance frequency analysis (Osstell Implant Stability Quotient (ISQ); Osstell AB, Gothenburg, Sweden). Cover screws were placed.

### Augmentation protocol

Venous blood (10 mL) was drawn from antecubital vein from each patient and centrifuged at 3000 rpm for 10 min using a bench-top centrifuge (Centrifuge 80 − 1) [31]. Platelet-rich fibrin (PRF) was prepared and mixed with xenograft particles (Bio-Oss^®^, Geistlich Pharma, Wolhusen, Switzerland) to graft the interpositional space and covered with HydroSorb™ Collagen Membrane (Zimmer Biomet, Warsaw, IN, USA) and primary closure was achieved.

### Postoperative care

Patients received standard postoperative medication, including antibiotics, anti-inflammatories, analgesics, and 0.2% chlorhexidine mouthwash. Follow-ups at 1, 3, and 7 days evaluated pain, swelling, and complications [33].

### Prosthetic phase

After four months, implants were uncovered and open-tray impressions taken. Screw-retained monolithic zirconia crowns were fabricated using CAD/CAM, inserted and torqued to 25 Ncm.

### Radiographic evaluation and accuracy assessment

Cone-beam computed tomography (CBCT) scans were taken at baseline prior to surgery (T0) and immediately postoperative (T1). Implant position accuracy was measured by superimposing T0 and T1 CBCT data evaluating the following parameters:

- Coronal deviation (mm).
- Apical deviation (mm).
- Angular deviation (°).
- All measurements were conducted by 2 blinded evaluators; inter-examiner reliability was assessed using intraclass correlation coefficient (ICC) [34].

### Accuracy analysis

Planned and actual implant positions were compared using a fully digital workflow based on CBCT data and 3D model superimposition. STL models were aligned and analyzed to calculate linear and angular deviations. The workflow utilized open-source software for segmentation, registration, and coordinate-based deviation analysis. Positional deviation calculations were based on basic Euclidean distance formulas between planned and actual implants position.

Coronal Deviation $$D_c\;=\;\sqrt{\left(X_{c,p}-X_{c,a}\right)^2\;+\;\left(Y_{c,p}-\;Y_{c,a}\right)^2\;+\;\left(Z_{c,p}-\;Z_{c,a}\right)^2}$$ 

Apical Deviation $$D_c\;=\;\sqrt{\left(X_{a,p}-X_{a,a}\right)^2\;+\;\left(Y_{a,p}-\;Y_{a,a}\right)^2\;+\;\left(Z_{a,p}-\;Z_{a,a}\right)^2}$$ 

Angular Deviation $$\theta\;=\;\arccos\;\left(\frac{V_p,\;V_a}{\left\|V_p\right\|\times\left\|V_a\right\|}\right)\;\times\;\frac{180}\pi$$ 

### Statistical analysis

All statistical analyses were conducted using SPSS software (version 22.0; IBM Corp, Armonk, NY, USA). To account for the clustering of implants within patients, a Linear Mixed Model (LMM) was used. Each patient was treated as a random effect and the augmentation group (ridge splitting vs. osteoperiosteal flap) was included as a fixed effect using the model structure: outcome ~ group + (1|patient). This method accounts for intra-patient correlation and provides unbiased estimates of group differences.

The LMM was selected over conventional methods such as t-tests or Mann–Whitney U tests, which assume independence of observations—an assumption violated when multiple implants are placed within the same patient. Results are reported as mean differences with 95% confidence intervals (CIs) and exact p-values. Statistical significance was set at (*p* < 0.05).

### Null hypothesis

The null hypothesis of this study was that there is no statistically significant difference in the 3D accuracy of implant placement between the guided ridge splitting and guided osteoperiosteal flap techniques.

## Results

A total of 18 patients (10 females, 8 males; mean age: 34.2 ± 6.1 years) were included in the study based on the previously mentioned inclusion and exclusion criteria. A total of 40 implant placements were analyzed, distributed between the ridge splitting group (*n* = 20) and the osteoperiosteal flap group (*n* = 20). All patients demonstrated uneventful postoperative healing, and no major complications such as infection, flap necrosis, or implant failure were recorded. The statistical comparison between the two groups focused on three primary deviation parameters: Three-dimensional (3D) coronal deviation, Three-dimensional (3D) apical deviation, and Three-dimensional (3D) angular deviation.

The analysis showed that 3D coronal deviation was higher in the ridge splitting group (1.1 ± 1.3 mm) compared to the osteoperiosteal flap group (0.6 ± 0.3 mm), with a mean difference of 0.5 mm (95% CI: −0.1 to 1.1; *p* = 0.103). Similarly, 3D apical deviation was (1.1 ± 1.6) mm in the ridge splitting group and (0.6 ± 0.3 mm) in the osteoperiosteal flap group, yielding a mean difference of 0.5 mm (95% CI: −0.2 to 1.2; *p* = 0.180). For 3D angular deviation, the ridge splitting group showed (3.8 ± 2.2°) compared to (3.5 ± 1.8°) in the osteoperiosteal flap group, with a mean difference of 0.2° (95% CI: −1.1 to 1.5; *p* = 0.720) (Table 1).

**Table 1:** Comparison of deviation parameters between ridge splitting and osteoperiosteal flap groups

Parameter	Ridge Splitting (*n* = 20) Mean ± SD	Osteoperiosteal Flap (*n* = 20) Mean ± SD	Mean Difference (95% CI)		*p*-value
3D Coronal Deviation (mm)	**1.1 ± 1.3**	**0.6 ± 0.3**		**0.5 mm (− 0.1 to 1.1)**	**0.103**^a^
3D Apical Deviation (mm)	**1.1 ± 1.6**	**0.6 ± 0.3**		**0.5 mm (− 0.2 to 1.2)**	**0.180**^a^
3D Angular Deviation (°)	**3.8 ± 2.2**	**3.5 ± 1.80**		**−0.2° (− 1.1 to 1.5)**	**0.720**^a^

^a^Not statistically significant (*p* > 0.05)

In all parameters, ridge splitting showed numerically higher deviations; however, none of the differences reached statistical significance, as all 95% confidence intervals crossed zero and *p*-values exceeded 0.05.

## Discussion

Static computer aided implant surgery (sCAIS) helps improve accuracy, makes the procedure more predictable and allows for better intraoperative surgical control. Studies have shown that static guides minimize implant placement errors in coronal, apical, and angular deviation compared to freehand techniques in partially edentulous patients [12, 16, 35].

Implant placement outcomes are influenced by deviations in (Standard Tessellation Language (STL) – Cone-beam computed tomography (CBCT) alignment, surgical guide design, sleeve length and tolerance, mucosal thickness, and 3D printing material dimensional stability, all of which have been shown to impact outcomes [17, 36, 37].

Our study accounted for these variables through cross-verification of STL-CBCT alignment, use of high-resolution stereolithographic (SLA) guides, and sleeveless surgical guide tubes to control vertical deviation.

The use of an entirely digital workflow — incorporating CBCT imaging, intraoral scanning virtual planning software (BlueSkyBio), and chairside stereolithographic (SLA).

3 dimensional (3D) printing — further minimized human error and allowed for fast and reliable surgical guide fabrication.

Previous research has shown that digital workflows often result in implant placement deviations that fall within acceptable clinical limits — generally defined as ≤ 2 mm at the entry point and ≤ 5° in angular deviation.

In their systematic review Tahmaseb et al. (2018) reported mean coronal deviations of 1.2 mm, mean apical deviation 1.4 mm, and mean angular deviation 3.5°. Our study achieved even greater accuracy, with mean coronal, apical, and angular deviations 1.1 ± 1.3 mm, 1.1 ± 1.6 mm, and 3.8 ± 2.2° in the ridge splitting group and 0.6 ± 0.3 mm, 0.6 ± 0.3 mm, 3.5 ± 1.8° in the osteoperiosteal flap group, supporting the precision of our guided approach [12]. (Table 1)

Our results are also consistent with those reported by D’haese et al. (2012), who evaluated the accuracy of mucosa-supported surgical guides in the treatment of fully edentulous maxillas. Their data showed average deviations of 0.91 mm at coronal entry, 1.13 mm at apex, and 2.6° in angular deviation. By comparison, our approach yielded comparable or superior results with improved linear precision [14].

Eftekhar Ashtiani et al. (2020), in their systematic review of static guide systems reported that the average values of deviation were 1.16 mm at the coronal level, 1.35 mm apically, and 3.43° in angular deviation. Compared to these results, the present study showed reduced deviations at both the coronal (1.1 ± 1.3 mm) (0.6 ± 0.3 mm) and the apical (1.1 ± 1.6) (0.6 ± 0.3 mm) points. Although our angular deviation was slightly higher at (3.8 ± 2.2°) and (3.5 ± 1.8°), it still fell within clinically acceptable limits. These findings further validate the consistency of our fully digital, dual-guide workflow for guided implant surgery [38].

Ridge splitting in narrow anterior ridges, is associated with technical difficulty and a higher risk of buccal plate fracture or inadequate expansion. Ridge splitting has traditionally been done with open-flap surgery and direct visualization, which can increase the risk of bone loss and soft tissue damage after the procedure [23–39]. In this study the osteotomy was guided using a digitally fabricated surgical guide and performed with piezoelectric device allowing for controlled bone expansion. The results were consistent with other findings that support the use of guided ridge expansion methods. A study by Mustafa et al. (2017) described successful implant placement and measurable ridge splitting using similar tools and techniques [40].

The clinical advantages match with the esthetic and biological principles of implant site development stated by Buser et al. (2004) [41]. The use of piezoelectric surgery device improved control in crestal osteotomy depth while minimizing thermal damage enabling micrometric, selective cutting of mineralized tissue while preserving surrounding soft tissues, a significant benefit in anatomically challenging sites like the anterior maxilla [42, 43]. Also, piezosurgery enabled mobility of the buccal plate, even within dense cortical bone, thus promoting atraumatic ridge splitting.

Surgical guide stability and underlying bone support are both directly related to improved accuracy in implant placement [44]. To ensure this we used a dual-guide technique in our protocol, dividing ridge splitting or osteoperiosteal flap elevation and implant placement into two separate stages. This method offered better surgical control and reduced the risk of cumulative surgical guide deformation, an issue reported in previous research using single surgical guide [14, 45].

Additionally, the combination of autologous platelet-rich fibrin (PRF) with xenograft to fill the interpositional created gap between the split bony segments enhanced wound healing in our patients by stimulating angiogenesis, promoting new bone formation, and improving soft tissue repair [46, 47].

Zhou et al. [36] reported that fixation of surgical guides, type of support (tooth, mucosa, bone), and the use of a flapless technique significantly impact angular and linear deviations. Our use of rigid, tooth- or bone-supported surgical guides and verification of passive fit reduced these sources of inaccuracy.

Beyond placement accuracy, our protocol addressed clinical challenges in narrow ridge augmentation in esthetically sensitive anterior maxilla by directly comparing the osteoperiosteal flap technique to ridge splitting technique.

The osteoperiosteal flap, despite historical origins in classical maxillofacial and orthopedic surgery, has never been applied in the context of guided implant surgery [48].

Our application of this flap in a digitally guided, piezosurgery assisted surgery signifies a new adaption of the technique.

In contrast to ridge splitting, which require significant flap reflection and risk the buccal plate’s stability through possible fracture or resorption, the osteoperiosteal flap preserves bone. Jensen et al. (2009) recorded a reduced occurrence of buccal bone loss in osteoperiosteal flap (1 out of 65) compared to ridge splitting (10 out of 65) during alveolar ridge splitting surgeries. This flap design also attained a high implant osseointegration rate of 92.5% [8]. Additional studies support its use for predictable horizontal and vertical ridge augmentation with lower risks [9]. In our hands, this technique allowed for better mobilization of the buccal plate while minimizing trauma.

Although the osteoperiosteal flap group showed numerically lower deviation values, these differences were not statistically significant (*p* > 0.05), and both techniques can be considered equally accurate within the limits of this study (Table 1).

These results are in line with earlier observations by Jensen et al. (2009) [8], who emphasized the role of osteoperiosteal flaps in maintaining buccal bone.

Finally to precisely evaluate deviation between the planned and postoperative implant positions, we used a digital workflow that included several software tools, each serving a specific role in the analysis process.

We started by STL export of planned implant positions using BlueSkyPlan (BlueSkyBio, LLC, Grayslake, IL, USA).

Then postoperative CBCT data (T1) were processed in 3D Slicer to segment and extract the actual implant geometries as STL files.

We then used MeshLab (Visual Computing Lab, ISTI–CNR, Pisa, Italy) to register the planned and postoperative implant models by selecting specific anatomical landmarks.

Then CloudCompare (Open-source; originally developed at Télécom Paris, France) was used to pinpoint coronal and apical locations, from which XYZ coordinates were extracted for further analysis.

These coordinates were then imported into Microsoft Excel (version 2019; Microsoft Corporation, Redmond, WA, USA), where spatial differences in coronal and apical deviations were calculated using geometric formulas that were based on basic Euclidean distance formulas, while angular deviations were calculated using the angle between the planned and actual implant in 3D space as described by Cassetta et al. [44].

**Fig. 6:**
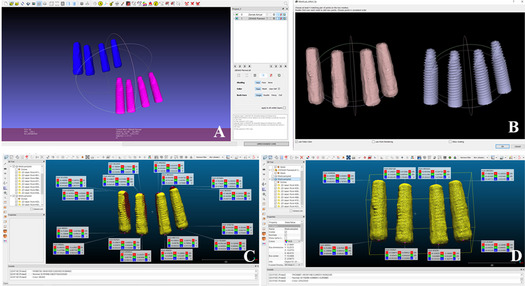
Superimposition workflow for planned and actual implant positions in a ridge splitting case. **A** Overlay of planned and actual implant positions. **B**, Anatomical landmarks used for superimposition. **C**, Comparison of planned and actual implant coordinates in X, Y, Z axes. **D**, Actual implant coordinate positions. Measured postoperatively using implant planning software

**Fig. 7:**
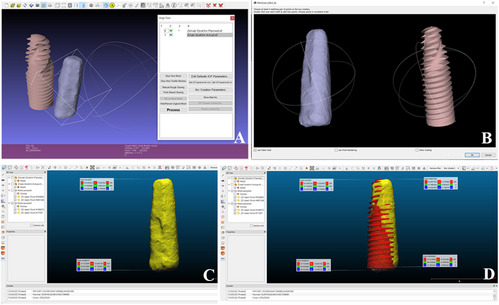
Superimposition workflow for planned and actual implant positions in an osteoperiosteal flap case. **A** Overlay of planned and actual implant positions. **B** Anatomical landmarks used for superimposition. **C** Actual implant coordinate positions. **D** Comparison of planned and actual implant coordinates in X, Y, Z axes. Measured postoperatively using implant planning software

This integrated software sequence maintained a consistent coordinate system throughout and enabled precise assessment of implant placement accuracy (Figures 6 and 7).

In summary, this randomized clinical trial demonstrated that both the guided ridge splitting and guided osteoperiosteal flap techniques yielded comparable and clinically acceptable accuracy for implant placement when used within a fully digital, dual-guide workflow. These results support the integration of digital planning with guided bone augmentation as a feasible and precise approach for treating atrophic maxillary ridges. To the best of our knowledge, this study represents a novel adaptation that may offer a minimally invasive alternative to traditional GBR techniques in select clinical scenarios. Future studies with larger sample sizes and long-term follow-up are recommended to validate these findings and assess clinical outcomes beyond accuracy.

### Limitations

This study had several limitations. First, it was conducted at a single academic center by a limited number of operators, which may introduce operator bias and limit generalizability.

Second, although accuracy was rigorously assessed using a validated digital workflow, only short-term outcomes were evaluated. Long-term clinical performance, including implant survival, peri-implant bone stability, and prosthetic success, was not within the scope of this study and remains to be investigated in future studies.

Third, while the sample size was statistically adequate for the primary outcome (accuracy), it may have been insufficient to detect smaller yet clinically meaningful differences between groups. Future multicenter studies with larger sample sizes and long-term follow-up are recommended to further validate these findings.

## Conclusion

This study demonstrated that both the guided ridge splitting and guided osteoperiosteal flap techniques, when implemented within a fully digital, dual-guide workflow, resulted in comparable and clinically acceptable accuracy for implant placement in narrow maxillary ridges. While minor numerical differences were observed between the two techniques, these were not statistically significant (*p* > 0.05). These findings support the use of either approach as a reliable and digitally driven method for achieving accurate implant placement following augmentation procedures in atrophic maxillae.

## Data Availability

The datasets used and/or analysed during the current study are available from the corresponding author on reasonable request.

## Abbreviations

CAD/CAM: Computer aided design/ computer aided manufacturing
CBCT: Cone-beam computed tomography
DICOM: Digital imaging and communications in medicine format
GBR: Guided bone regeneration
ICC: Intraclass correlation coefficient
ISQ: Implant stability quotient
PRF: Platelet rich fibrin
RCT: Randomized clinical trial
sCAIS: Static computer-aided implant surgery
SLA: Stereolithography
STL: Standard tessellation language
